# Supplementary material for: Characterization of an intracellular humanized single-chain antibody to matrix protein (M1) of H5N1 virus
Source: PLoS One. 2022 Mar 31;17(3):e0266220. doi: 10.1371/journal.pone.0266220 (PMC8970388; doi:10.1371/journal.pone.0266220)
Supplement: S1 File — (ZIP) [file pone.0266220.s001.zip › attached file/C. Purification of PTD-GFP fusion protein and determination of its transduction efficiency.pdf]

PTD-GFP 融合蛋白的纯化及其转导效率测定

徐艳玲[1],岳玉环[1],张国利[1],吴广谋[1],田　园[1],张培培[1],付玉和[2],赵　鑫[2],侯天全[2]
(1.军事医学科学院 军事兽医研究所,长春 130122;2.吉林农业大学 生命科学学院,长春 130118)

中图分类号:S813.3　　文献标识码:A　　文章编号:1004-7034(2016)01-0025-05
DOI:10.13881/j.cnki.hljxmsy.2016.0007

关键词:蛋白转导域(PTD);绿色荧光蛋白(GFP);融合蛋白;纯化;转导效率

摘　要:为了纯化 PTD-GFP 融合蛋白,测定融合蛋白转导效率,研究融合蛋白在 HeLa 细胞中的转导效率与浓度的关系,试验诱导表达军事兽医研究所六室保存的含有 pET-20b-PTD-GFP 重组质粒的 BL21 表达菌,超声破碎,取上清液通过多个层析介质得到高纯度融合蛋白,将纯化的蛋白加入到体外培养的 HeLa 细胞中,在荧光显微镜下观察 PTD-GFP 的转导情况,用 Infinite® F500 多功能酶标仪检测荧光强度,分析融合蛋白浓度对转导效率的影响。结果表明:纯化的重组蛋白纯度达到 90%。原浓度蛋白和 1/2 原蛋白浓度 24 h 后转导效率都达到 100%。通过统计学软件得到转导效率与 PTD-GFP 融合蛋白浓度之间的线性关系,回归系数为 0.151 456,相关系数为 0.954。说明融合蛋白纯度较高,其转导效率对蛋白浓度具有依赖性,在一定浓度范围内随着浓度增加转导效率增加。

Purification of PTD-GFP fusion protein and determination of its transduction efficiency

XU Yanling[1], YUE Yuhuan[1], ZHANG Guoli[1], WU Guangmou[1], TIAN Yuan[1], ZHANG Peipei[1], FU Yuhe[2], ZHAO Xin[2], HOU Tianquan[2]
(1. Institute of Military Veterinary, Academy of Military Medical Sciences, Changchun 130118, China;
2. College of Life Sciences, Jilin Agricultural University, Changchun 130118, China)

Keywords: PTD; green fluorescent protein(GFP); fusion protein; purification; transduction efficiency

Abstract: To purify the PTD-GFP fusion protein and determine its transduction efficiency and study the relation between its transduction efficiency and concentration in the HeLa cells, the BL21 recombinant bacteria containing a recombinant plasmid pET-20b-PTD-GFP presenved in the sixth laboratory of the Institute of Military Veterinary, was induced for expression. The recombinant bacteria were disrupted by ultrasonic instrument. The high-purity fusion protein in the supernatant was obtained by a plurality of chromatographic media. The purified fusion protein was added into the HeLa cells cultured in vitro, and then the transduction of PTD-GFP was observed by a fluorescence microscope. The fluorescence intensity was detected by a multifunctional microplate reader Infinite® F500, and then the effect of fusion protein concentration on transduction efficiency was analyzed. The results showed that the purity of purified fusion protein reached 90%, and the transduction efficiencies of the original protein concentration reached 100% in accord with one-half original protein concentration after 24 h. There is a linear relation between transduction efficiency and concentration of PTD-GFP by the analysis of the statistical software, and the coefficient of regression and correlation coefficient were 0.151 456 and 0.954, respectively. The results indicate that PTD-GFP fusion protein reached a high-purity level, and the transduction rate of fusion protein has a dependency on the protein concentration, and it increases with the increase of the protein concentration within a certain range of concentrations.

CPPs(cell penetrating peptides)是一类具有跨膜能力、含有 5~40 个氨基酸残基的短肽。这类短肽的出现为药物分子进入细胞发挥治疗作用带来希望。1988 年,M. Green 等[1]首次报道人工合成 HIV-1 反转录激活因子 TAT(transactivator of transcription)全长蛋白可以通过 HeLa 细胞膜,具有跨细胞膜特性,随后开启了众多实验室对 TAT 蛋白转导功能的相关研究。TAT 蛋白是目前 CPPs 中研究最热的蛋白之一。TAT 蛋白含有 N-末端激活区、半胱氨酸富集区、中心区、碱性氨基酸富集区和谷氨酰胺富集区[2-4] 5 个区域,其中含有 11 个氨基酸(YGRKKRRQRRR)的碱性氨基酸富集区是其转导的核心区域 PTD(Protein transduction domain)。有研究表明,PTD 的转导能力不比全长 TAT 序列差[5-6],核心区的精氨酸在其转导中起关键作用[7],所携带的正电荷对其跨膜功能有重要影响[8-9]。目前,许多研究人员对于 TAT 蛋白转导效率的高低还存在较大争议,本次研究利用绿色荧光蛋白(green fluorescent

收稿日期:2015-04-23;修回日期:2015-10-23
基金项目:吉林省重点科技攻关项目(20130206012YY)
作者简介:徐艳玲(1990-　),女,硕士研究生,研究方向为蛋白质纯化工艺,1073536810@qq.com.
通信作者:岳玉环(1963-　),女,副研究员,博士,研究方向为基因工程及生物制药,yhyue2013@163.com.

(C)1994-2019 China Academic Journal Electronic Publishing House. All rights reserved.　http://www.cnki.net

protein,GFP)作为报告蛋白,在大肠杆菌中表达、纯化 PTD－GFP 融合蛋白,检测 TAT 蛋白中的核心肽段 PTD 携带 GFP 蛋白对 HeLa 细胞膜的转导情况以及该蛋白浓度与转导效率间的相关性,为下一步利用 PTD 蛋白携带外源蛋白进入细胞内发挥治疗作用提供相应的研究基础。

1 材料与方法

1.1 细菌及细胞

含 pET－20b－PTD－GFP 重组质粒的 BL21 表达菌和 HeLa 细胞,均由军事医学科学院军事兽医研究所六室保存。

1.2 主要试剂和仪器

Phenyl－HP、Sephadex G25 和 Q Sephadex Fast Flow,均购自美国 Pharmacia Biotech 公司;BCA 蛋白浓度试剂盒、RIPA 细胞裂解液,购自碧云天生物技术研究所;1640 培养基、双抗、Hepes,购自普洛麦格(北京)生物技术有限公司;血清、96 孔细胞培养板,购自上海百研生物科技有限公司;96 孔黑色微孔板,购自上海精睿生物科技发展有限公司;奥林巴斯 IX71 显微镜、Infinite® F500 多功能酶标仪,购自上海迪奥生物科技有限公司。

1.3 重组蛋白的纯化

1.3.1 硫酸铵梯度沉淀粗提目的蛋白　将表达的融合蛋白菌用不同浓度的硫酸铵进行梯度沉淀,选取最高沉淀杂蛋白、而目的蛋白不被沉淀的浓度梯度,以此来粗提目的蛋白。称取 1 g 菌,用 20 mmol/L Tris－Cl(pH 值为 8.0)10 mL 溶解,超声裂解(功率 400 W,工作时间 5 s,间歇 9 s,共 30 min),4 ℃、9 600×g 离心 30 min;取上清液加入硫酸铵至浓度为 0.2 mol/L,4 ℃放置 30 min;4 ℃、11 000 r/min 离心 30 min;取上清液继续用 0.4,0.6,0.8,1,1.5,2,2.5 mol/L 梯度硫酸铵沉淀,然后每步沉淀以等体积 20 mmol/L Tris－Cl(pH 值为 8.0)重悬,取样进行 12% SDS－PAGE 分析,确定最适粗提目的蛋白的硫酸铵浓度。

1.3.2 Phenyl－HP 疏水层析　用 30 mL A1 液[20 mmol/L Tris－Cl(pH 值为 8.0),1.25 mol/L 硫酸铵]溶解 3 g 菌,按如上处理方式超声裂解。用 A 液平衡层析柱,以 2 mL/min 流速上样,用 B1－1 液[20 mmol/L Tris－Cl(pH 值为 8.0),0.5 mol/L 硫酸铵]、B1－2 液[20 mmol/L Tris－Cl(pH 值为 8.0),0.2 mol/L 硫酸铵]进行阶段洗脱,收集蛋白峰,进行 12% SDS－PAGE 电泳。

1.3.3 Sephadex G25 和 Q 阴离子交换层析　用 A2 液[20 mmol/LTris－Cl(pH 值为 8.0)]平衡 Sephadex G25 脱盐柱,以 15 mL/min 流速上样,然后用 A2 液进行洗脱,收集蛋白。Q 强阴离子交换层析柱用 A2 液平衡,以 1.5 mL/min 流速上样,用 B2 液[20 mmol/L Tris－Cl(pH 值为 8.0),0.2 mol/L NaCl]洗脱,收集蛋白峰,进行 12% SDS－PAGE 电泳分析,用 BCA 试剂盒测定蛋白浓度。

1.4 PTD－GFP 融合蛋白不同浓度的转导效应

1.4.1 细胞的培养　HeLa 细胞用含 10% 胎牛血清的 1640 培养基进行培养,置于 37 ℃、5% CO_2 培养箱中。转导前以每孔 5×10^3 个细胞接种于 96 孔细胞培养板,24 h后添加 PTD 融合蛋白。

1.4.2 细胞内融合蛋白的荧光检测　用无血清 1640 稀释目的蛋白,蛋白初始浓度为 380 μg/mL。先将 PTD－GFP 融合蛋白以原浓度加入 96 孔细胞培养板中,再将 PTD－GFP 融合蛋白在以原浓度 1/2(即 190 μg/mL)和 1/3(即 126.67 μg/mL)的基础上进行倍比稀释,共计 17 个浓度,每个浓度做 3 个复孔,最后 3 列只添加无血清的 1640 培养基作为阴性对照。24 h 后,用 37 ℃生理盐水洗板 3 次,在荧光显微镜下观察蛋白的转导情况。

1.4.3 细胞内融合蛋白荧光强度的检测　在观察后的细胞板中每孔加入 100 μL 细胞裂解液,将裂解的细胞转移至 96 孔黑色微孔板,3 000×g 离心 30 min,在激发光波长为 485 nm 、发射光波长为 533 nm 的条件下用多功能酶标仪进行检测。转导效率＝(转导组荧光强度－非转导组荧光强度)/(原蛋白浓度荧光强度－非转导组荧光强度)。

2 结果

2.1 硫酸铵梯度沉淀结果

12% SDS－PAGE 分析结果表明,2 mol/L 硫酸铵会使少量目的蛋白发生沉淀,1.5 mol/L 硫酸铵的条件下可以除去大部分杂蛋白(见图 1),因此最适合的硫酸铵浓度为 1.5 mol/L。

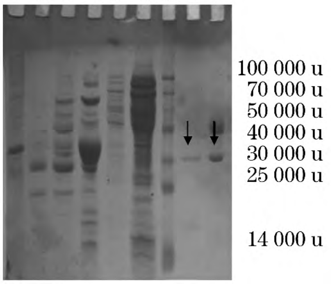

1～8. 分别为 0.2,0.4,0.6,0.8,1,1.5,2,2.5 mol·L^{-1} 硫酸铵沉淀产物;M. 蛋白 Marker。

图 1　不同浓度硫酸铵沉淀去除杂蛋白的 SDS－PAGE 分析

Fig.1　SDS－PAGE analysis of using different concentration of ammonium sulfate precipitation to remove contaminating proteins

2.2 Phenyl－HP 疏水层析结果

(C)1994-2019 China Academic Journal Electronic Publishing House. All rights reserved. http://www.cnki.net

12% SDS - PAGE 分析结果表明，样品经过 Phenyl - HP 疏水层析，用 B1 - 1 液进行截留洗脱，大部分杂蛋白被除去，目的蛋白并未被洗掉，当换用 B1 - 2 液洗脱时收集到含有大量目的蛋白的样品，见图 2。

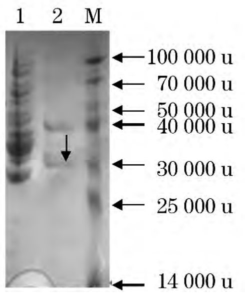

1. B1 - 1 液洗脱; 2. B1 - 2 液洗脱; M. 蛋白 Marker。

图 2 Phenyl - HP 层析产物的 SDS - PAGE 分析

Fig. 2 SDS - PAGE analysis of the purified product by Phenyl - HP chromatography

2.3 Q 阴离子交换层析结果

12% SDS - PAGE 分析结果表明，经过疏水层析收集到的样品通过 Q 柱的纯化及浓缩得到了纯度较高的目的蛋白，蛋白的纯度达 90% 以上(见图 3)，BCA 蛋白浓度试剂盒测定结果为 12.67 μmol/L。

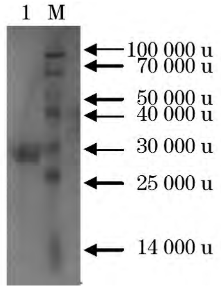

1. 目的蛋白; M. 蛋白 Marker。

图 3 离子交换层析产物的 SDS - PAGE 分析

Fig. 3 SDS - PAGE analysis of the purified product by ion - exchange chromatography

2.4 细胞转导效率

荧光显微镜下观察结果表明，细胞在激发光下发出绿色荧光，1/2 原蛋白浓度和原蛋白浓度转导效率都达到 100%，二者的荧光强度经统计学分析不具有差异性，随着蛋白浓度的降低，转导率随之降低，见 257 页彩图 4。由 257 页彩图 4C 可以明显地观察到融合蛋白转导到细胞内部。

图 5 为统计学软件处理得到的线性回归结果，预测转导效率方程为 =0.091 874 +0.151 456(PTD - GFP 蛋白浓度)，可以看到回归系数(0.151 456) 不为 0(P <0.001)，表明浓度在 0 ~ 6.33 μmol/L 范围内，每增加 1 μmol/L，预测转导效率将增加 15.1%，相关系数为 0.954。

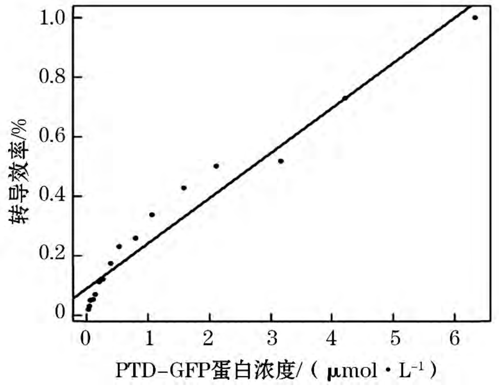

图 5 不同蛋白浓度的转导效率

Fig. 5 The transduction efficiencies of protein under different concentrations

3 讨论

尽管 TAT 蛋白跨膜和基因调控机制至今尚未研究清楚，但是并不影响研究人员对其的利用。近年来，对 TAT 蛋白的转导作用被用于多种研究[10-13]，而不同的研究 TAT 蛋白对其影响不同。TAT(PTD) 同热休克蛋白 27 形成的融合蛋白可以保护上皮细胞减少紫外损伤[10]，而 M. C. Shin 等[11] 利用 TAT 蛋白的转导特性与白树毒素构成融合蛋白治疗癌症，但却降低了白树毒素对细胞的毒性，这恰恰也证明了 TAT 蛋白的安全性。TAT 蛋白安全性高、细胞破坏性小、靶标无特异性，具有携带外源分子进入细胞内的优势，促使了研究人员对其的研究利用，然而 TAT 蛋白转运效率高低仍然是一个模糊的概念。A. A. Baoum 等[14] 将携带质粒的 TAT 与聚乙酰亚胺(PEI，Polyemylenirnine) 在转导能力方面进行了对比和研究，确实发现其转染能力比 PEI 快得多，在 A549 细胞中前者是后者的 25 倍。但由于 TAT 的转导效率在不同程度上受到如分子大小、结构、性质、连接方式、内外环境等因素的影响，因此对其转导效率的准确界定是很困难的。

本研究利用 GFP 作为报告蛋白和观察指标，是非常直观明显的，利用多功能酶标仪可以很好地反映 GFP 的量。张垲等[15] 的研究表明，荧光蛋白浓度与荧光强度之间有良好的线性关系。本研究利用此种关系将荧光强度转化成PTD - GFP 的转导效率，从而直观地反映了蛋白浓度与转导效率的关系。

本研究针对 TAT 核心区 PTD 与 GFP 构建的融合蛋白研究其转导效率与浓度的关系，发现融合蛋白转导效率在一定浓度范围内随着浓度增大转导效率增加，且具有线性关系，对其转导效率可以进行预测。A. Roncador 等[16] 的研究证实，TAT - GFP 融合蛋白转导大鼠皮质神经元具有浓度依赖性。本研究 PTD - GFP 转导效率试验也确实证明了此结论。

(C)1994-2019 China Academic Journal Electronic Publishing House. All rights reserved. http://www.cnki.net

本研究以基因融合的形式构建了 PTD－GFP 融合蛋白,并检测了 PTD 携带 GFP 蛋白穿过细胞膜的情况,探索了融合蛋白浓度与转导效率之间的相关性,为后期的研究工作奠定了基础,也为其他研究者提供了相关资料。

参考文献:

[1] GREEN M, LOEWENSTEIN P M. Autonomous functional domains of chemically synthesized human immunodeficiency virus tat trans－activator protein [J]. Cell, 1988, 55(6): 1179－1188.

[2] 杨永臣,袁崇刚,李荣秀. Tat 蛋白及其内化作用 [J]. 生命的化学,2001,21(4):265－268.

[3] 艾菁,王丽梅,夏威,等. Tat 蛋白结构与功能的研究进展 [J]. 细胞与分子免疫学杂志,2005,21(Suppl): 133－135.

[4] 尹锐,郝飞. 穿膜肽 HIV Tat 蛋白的研究进展 [J]. 免疫学杂志,2005,21(3): 77－81.

[5] 郭爱华,刘志锋,孙学刚,等. 一种基于 HIV－1 TAT 蛋白质转导结构域细胞内转导系统的成功改建 [J]. 南方医科大学学报,2006,26(5):545－548.

[6] 李欢,胡晓梅,陈志瑾,等. TAT－EGFP 融合蛋白的表达纯化及穿膜活性的研究 [J]. 免疫学杂志,2008,24(6):630－633.

[7] 吴永红,张成岗. HIV－1 TAT 蛋白转导肽的研究进展 [J]. 中国生物工程杂志,2010,30(10):66－73.

[8] KOPPELHUS U, SHIRAISHI T, ZACHAR V, et al. Improved cellular activity of antisense peptide nucleic acids by conjugation to a cationic peptide－lipid (CatLip) domain [J]. Bioconjug Chem, 2008,19(8):1526－1534.

[9] ZHANG X K, LI Y, CHENG Y N, et al. Tat PTD－endostatin: A novel anti－angiogenesis protein with ocular barrier permeability via eye－drops [J]. Biochim Biophys Acta, 2015,1850(6):1140－1149.

[10] LIU L, YU R J, SHI Y H, et al. Transduced protein transduction domain linked HS P27 protected LECs against UVB radiation－induced damage [J]. Exp Eye Res,2014,120:36－42.

[11] SHIN M C, ZHAO J W, ZHANG J, et al. Recombinant TAT－gelonin fusion toxin: synthesis and characterization of heparin/protamine－regulated cell transduction [J]. J Biomed Mater Res A, 2015, 103(1):409－419.

[12] SHIN M C, ZHANG J, MIN K A, et al. Combination of antibody targeting and PTD－mediated intracellular toxin delivery for colorectal cancer therapy [J]. J Control Release,2014,194:197－210.

[13] NI Y W, YU J Y, XU J P, et al. Enhanced delivery of human growth hormone across cell membrane by Tat－PTD [J]. Endocrine, 2014, 46 (1):138－147.

[14] BAOUM A A, MIDDAUGH C R, BERKLAND C. DNA complexed with TAT peptide and condensed using calcium possesses unique structural features compared to PEI polyplexes [J]. Int J Pharm, 2014, 465(1/2):11－17.

[15] 张垲,余冰菲,陈瑞川,等. 荧光定量检测细胞绿色荧光蛋白技术的建立与应用 [J]. 厦门大学学报(自然科学版),2008,47(增刊2):264－267.

[16] RONCADOR A, OPPICI E, MONTIOLI R, et al. TAT－Mediated delivery of human alanine: glyoxylate aminotransferase in a cellular model of primary hyperoxaluria type I [J]. Int J Peptide Res Therap, 2013, 19 (2):175－184.

(009)

(上接第 24 页)

Study on the effects of different concentrations of dairy cow mammary epithelial cells co－cultured with dairy cow umbilical cord mesenchymal stem cells on the secretion of several cytokines

WANG Liwen, SHAO Wei, ZHAO Yankun, LI Yang, LIN Jing, YU Xiong

(Xinjiang Key Laboratory of Nutrition of Herbivores for Meat and Milk Production, College of Animal Science, Xinjiang Agricultural University, Urumqi 830052, China)

Keywords: umbilical cord mesenchymal stem cell; mammary epithelial cell; cytokine; epidermal growth factor (EGF); basic fibroblast growth factor (bFGF); transforming growth factor (TGF); hepatocyte growth factor (HGF)

Abstract: To study the effects of different concentrations of dairy cow umbilical cord mesenchymal stem cells (UC－MSCs) co－cultured with dairy cow mammary epithelial cells (DCMECs) on cell proliferation and cell adherence rate in the co－culture system, and explore the mechanism of action by detecting cell proliferation－related cytokines. The P3 generation of UC－MSCs and P3 generation of DCMECs were randomly mixed for culture according to different ratios of concentrations, including 1:1, 1:2, 1:3, 1:4, 1:5, 1:10, 1:50, 1:100, 1:1 000, 2:1 etc. meanwhile, the pure－culture groups for UC－MSCs and DCMECs were established as the control group. The supernatants were extracted to detect the secretion levels of epidermal growth factor (EGF), basic fibroblast growth factor (bFGF), transforming growth factor α (TGF－α), transforming growth factor β (TGF－β), and hepatocyte growth factor (HGF) at 0 h, 24 h, 48 h, 72 h, 96 h, 120 h and 144 h, respectively. The results showed that the secretion levels of EGF, HGF, and bFGF were highest at 72h after the UC－MSCs and DCMECs were co－cultured with different concentrations according to different ratios of concentrations, and the group with a concentration of 1:2 was significantly higher ($P<0.05$) than the control group; the secretion levels of TGF－α and TGF－β were lowest at 72 h, and the group with a concentration of 1:2 was significantly lower ($P<0.05$) than the control group. The results indicate that the UC－MSCs co－cultured with DCMECs can improve the secretion levels of EGF, HGF, and bFGF, and reduce the secretion levels of TGF－α and TGF－β. (009)

(C)1994-2019 China Academic Journal Electronic Publishing House. All rights reserved. http://www.cnki.net

彩 图

· 试验研究 ·

鸡卵清蛋白基因启动子克隆及其活性的检测

（作者郭　政 等，正文见第 5－7，11 页）

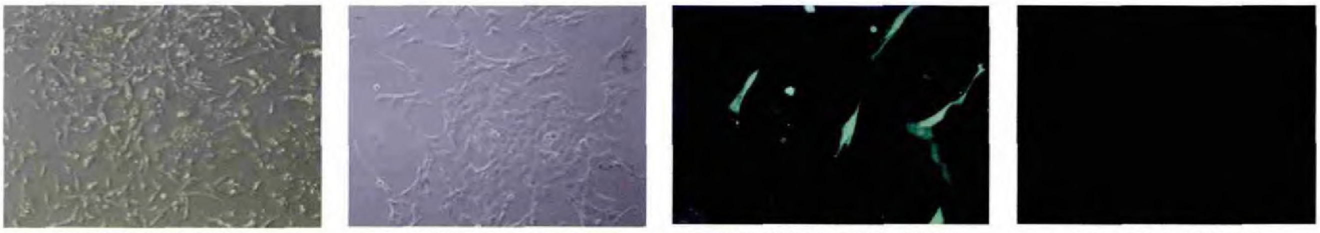

A 传代培养的输卵管上皮（50×）　B 传代培养的成纤维细胞（50×）　C 转染后的输卵管上皮细胞（200×）　D 转染后的成纤维细胞（200×）

图 4　转染 pAcGFP1－5OV 的鸡输卵管上皮细胞和成纤维细胞的 GFP 表达

Fig. 4　The expressions of green fl uorescent protein of chicken oviduct epithelial cells and fibroblasts transfected by pAcGFP1－5OV

· 试验研究 ·

不同浓度奶牛乳腺上皮细胞和奶牛脐带间充质干细胞共培养对几种细胞因子分泌的影响研究

（作者王立文 等，正文见第 21－24，28 页）

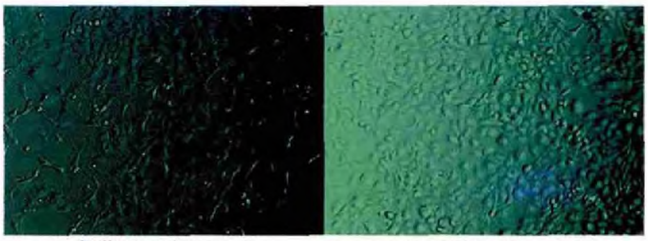

A 复苏后P1代BMECs（可以见到细胞周围有少许成纤维样细胞）　B 复苏后P2代BMECs（可见细胞之间紧密排列，呈鹅卵石样）

图 1　复苏后的 BMECs 形态（×40）

Fig. 1　The cell morphology of BMECs after recovery（×40）

· 试验研究 ·

PTD－GFP 融合蛋白的纯化及其转导效率测定

（作者徐艳玲 等，正文见第 25－28 页）

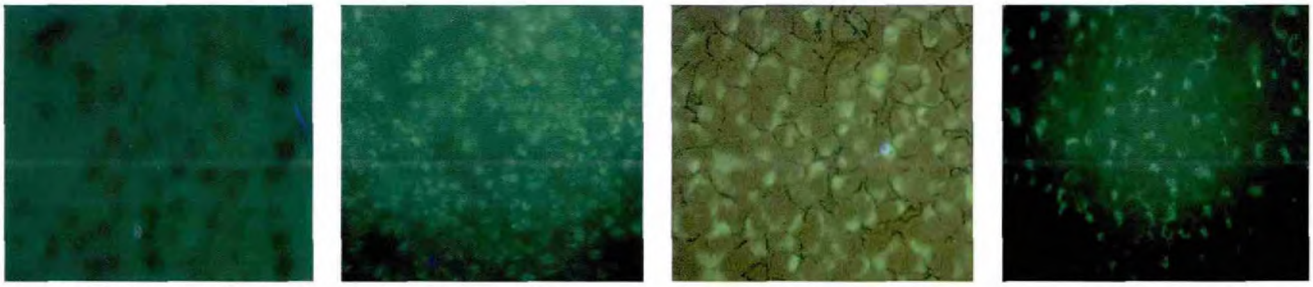

A 未转导的HeLa细胞（×10）　B 1/2原蛋白浓度转导的HeLa细胞（×10）　C 1/2原蛋白浓度转导的HeLa细胞（×40，半通光）　D 1/4原蛋白浓度转导的HeLa细胞（×10）

图 4　荧光显微镜观察的转导融合蛋白 24 小时后及未转导蛋白的 HeLa 细胞的荧光结果

Fig. 4　The fluorescent results of the fusion protein transduced after 24 h and non－transduced in the HeLa cells observed by the fluorescence microscope

(C)1994-2019 China Academic Journal Electronic Publishing House. All rights reserved.　http://www.cnki.net
