# Supplementary figures and images for: Characterization of an intracellular humanized single-chain antibody to matrix protein (M1) of H5N1 virus
Source: PLoS One. 2022 Mar 31;17(3):e0266220. doi: 10.1371/journal.pone.0266220 (PMC8970388; doi:10.1371/journal.pone.0266220)

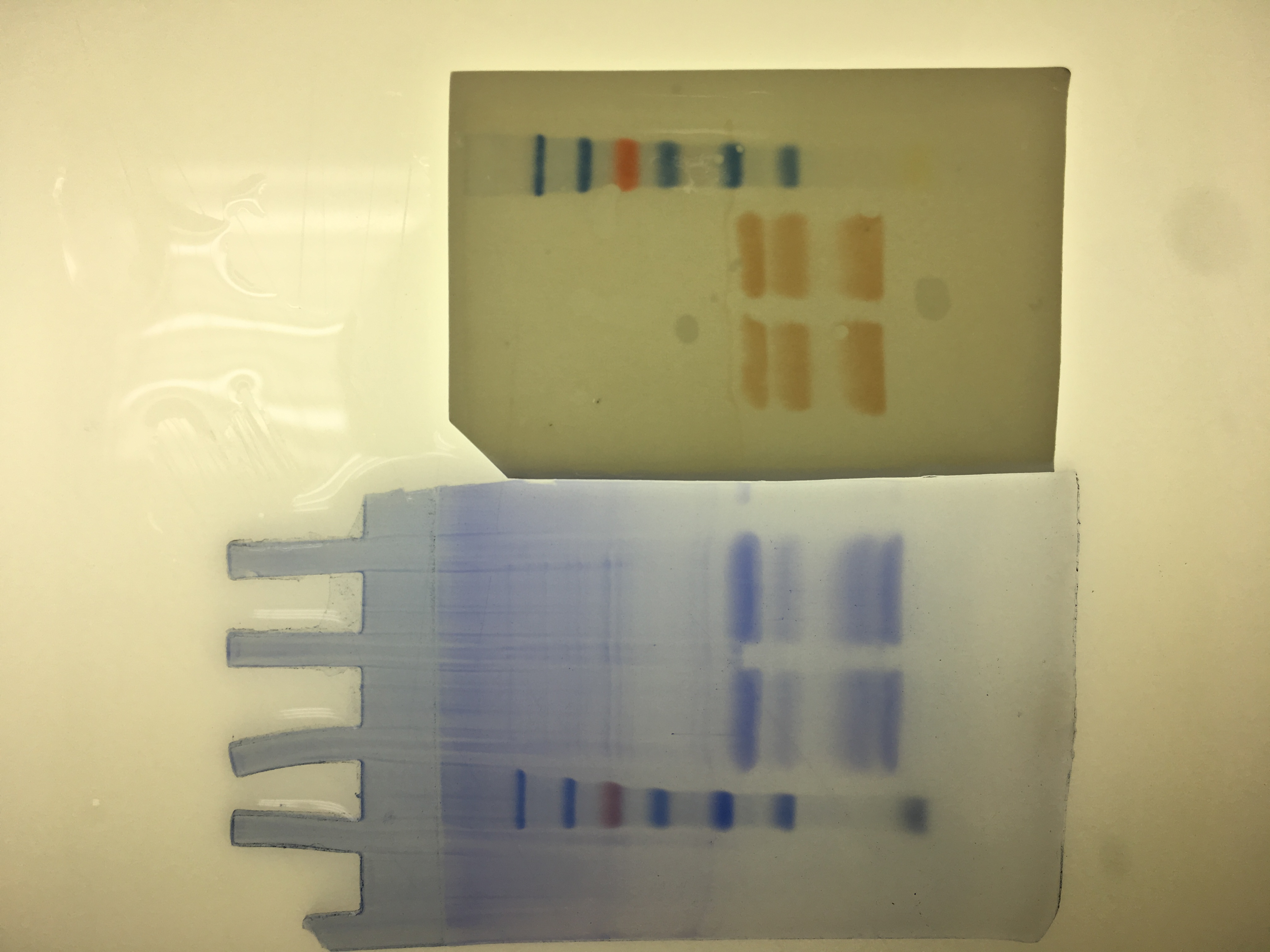

Supplement: S1 File — (ZIP) [file pone.0266220.s001.zip › attached file/D.S1 _ raw _ images.jpg]
