# Supplementary material for: Characterization of an intracellular humanized single-chain antibody to matrix protein (M1) of H5N1 virus
Source: PLoS One. 2022 Mar 31;17(3):e0266220. doi: 10.1371/journal.pone.0266220 (PMC8970388; doi:10.1371/journal.pone.0266220)
Supplement: Attachment — Submitted filename: Plosone_comments.docx [file pone.0266220.s002.docx]

Author Sun et. al., provides well characterized humanized antibodies against M1 protein, which showed excellent anti-influenza activity as shown by hemagglutination assay and found out that the single amino acid change may alter the binding. Here are my comments:

**Abstract section:**

1- Change the sentence to “cDNA of the H5N1 virus as a template; the M1 protein was then expressed and purified” to ‘cDNA of the H5N1 virus as a template, expressed in bacterial expression system (name the bacteria used) and purified.’
2- Space in 300 TCID50 line no 09; against the; line no 12.
3- Please use correct grammar and sentences in writing.

**Method section:**

1- First para line-3, provide the reference.
2- If virus was used in the study, please provide, the preparation method, stock concentration estimation etc.
3- Whether PCR amplicon of M1 was PCR/gel purified and any restriction enzymes were used to ligate into bacterial vector.
4- Please provide purification method for M1 protein
5- Please re-write this long sentence ‘Next, the Tomlison I+J phage antibody library was added and diluted with 2% milk/PBS to a titer of 1.0 ×1013; 100 μL was added to each well, and the liquid was incubated with vigorous shaking at room temperature for 60 min.’
6- WB analysis paragraph, used probed instead of ‘blotted’
7- Add extension time of PCR in Expression and Purification of TAT-HuScFv paragraph
8- First line of Hemagglutination Inhibition Analysis of Anti-M1-HuScFv and TAT-HuScFv ‘Digested MDCK cells’ with which enzyme?
9- Line 1-2; page no 12; please highlight or bold the changed amino acid in the small peptides.
10- Provide the role of GFP in this construct pET 28 TAT- HuScFv.

**Results section:**

Please provide a full blot of figure-2B.

In **discussion**, please provide the comparison of anti M1-TAT-HuScFv and previously published humanized antibodies against M1 protein.
